# Supplementary figures and images for: Characterization of occult hepatitis B in high-risk populations in Kenya
Source: PLoS One. 2020 May 28;15(5):e0233727. doi: 10.1371/journal.pone.0233727 (PMC7255601; doi:10.1371/journal.pone.0233727)

A

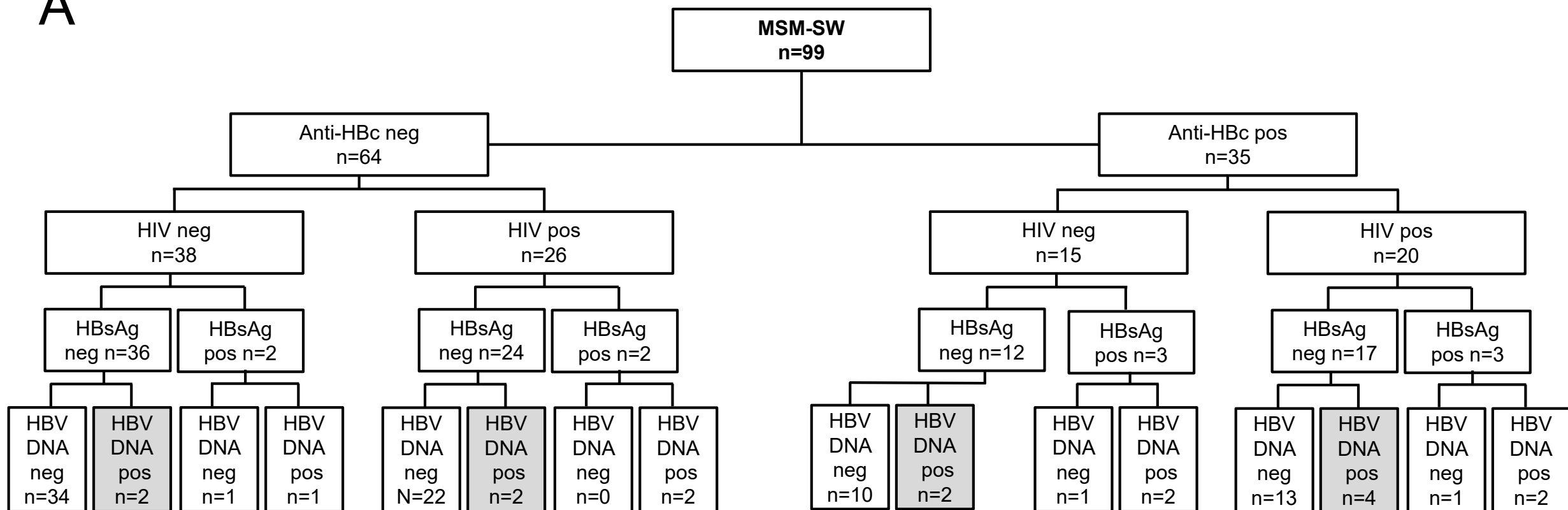

B

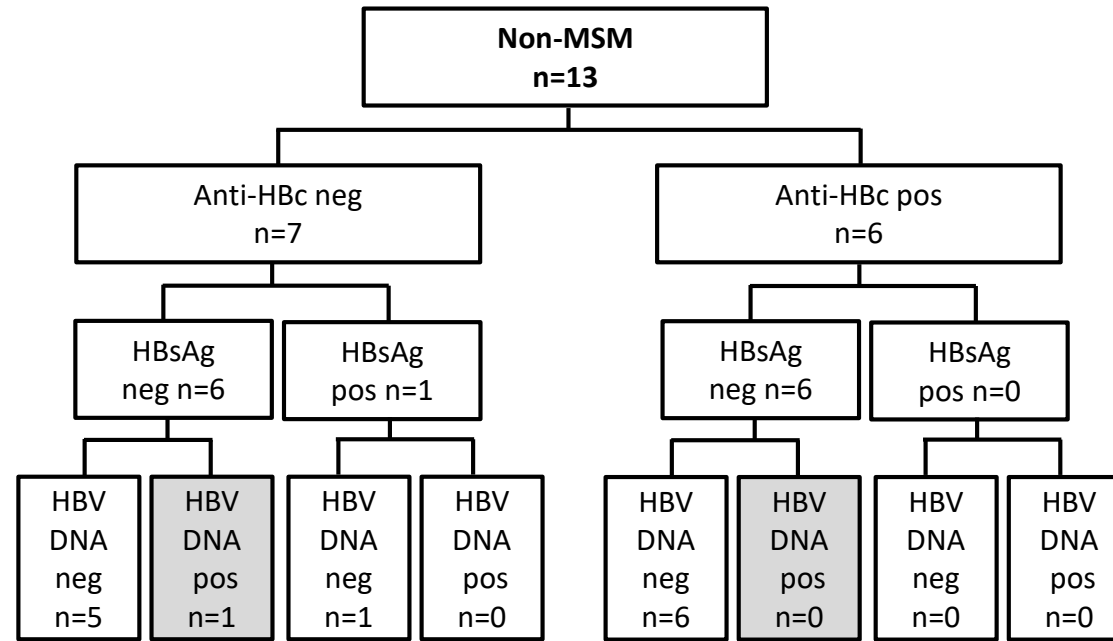

C

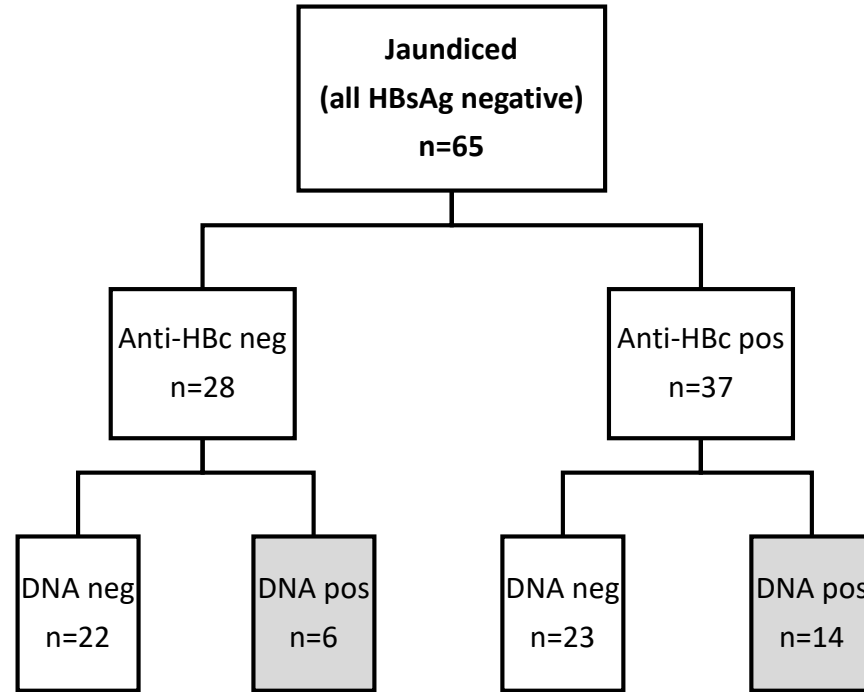

Supplement: S1 Fig — Shaded boxes denote OBI positive results. The total number of specimen results or specimens tested is noted within the box. MSM-SW specimens (A), non-MSM specimens (B), jaundiced patient specimens (C). HIV-reactivity results were determined during the original cohort study [20]. neg, Negative; pos, Positive. (PDF) [file pone.0233727.s001.pdf]
