# Supplementary material for: Characterization of occult hepatitis B in high-risk populations in Kenya
Source: PLoS One. 2020 May 28;15(5):e0233727. doi: 10.1371/journal.pone.0233727 (PMC7255601; doi:10.1371/journal.pone.0233727)
Supplement: S1 Table — (PDF) [file pone.0233727.s002.pdf]

S1 Table. HBsAg-coding region sequences included in phylogenetic analysis, by cohort or reference.

MSM-SW[a] OBI	MSM-SW HBsAg +	non-MSM OBI	Jaundiced OBI	GenBank reference[b] Kenyan sequence	non-Kenyan sequence
HY0016 (MK487155)	HY0038 (MK487146)	IMM0062 (MK487148)	EMB220 (MK487135)	A_JX154582	A1_AB116084_Bangladesh
HY0604 (MK487150)	HY0128 (MK487145)		EMB6B (MN972524)	A1_KP168425	A1_AB116087_India
SWM7256 (MK487151)	HY0479 (MK487149)		EMB7 (MK487137)	A1_KP168427	A1_AB116088_Nepal
	IMM0049 (MK487147)		KN113 (MK487153)	A1_KP168428	A1_AB116093_Philippines
	SWM2487 (MK487154)		KN120 (MK487139)	A1_KP168429	A1_AB453986_Japan
	SWM4590 (MK487133)		KN126 (MK487143)	A1_KP168430	A1_AY233277_S_Africa
	SWM10397 (MK487134)		KN160 (MK487142)	A1_KP168431	A1_AY934771_Somalia
			KN168 (MK487138)	A1_KP168432	A1_AY934772_Uganda
			KN194 (MK487140)	A1_KP168433	A1_AY934773_Tanzania
			KN88 (MK487136)	A1_KP168434	A1_DQ020002_Congo
			KSM583 (MK487152)	A1_KP168435	A1_FJ692587_Haiti
			KSM585 (MK487144)	A_KR816101	A1_FM199974_Rwanda
			KSM590 (MK487141)	A_KR816107	A1_HM535205_Zimbabwe
				A_KR816110	A1_U87742_S_Africa
				A_KR816115	A2_AB116076_USA
				A_KR816116	A2_AB116078_Japan
				A_KR816117	A2_AB453983_Japan
				A_KR816122	A2_AF297624_S_Africa
				A_KR816123	A2_AJ344115_France
				A_KR816125	A2_AY034878_USA
				A_KR816128	A2_GQ184324_S_Africa
				A_KR816129	A2_KP234051_Belgium
				A_KR816131	A2_KY003230_Eurohep
				A_KR816132	A2_KY382410_Argentina
				A_KR816133	A2_Z35717_Poland
				A_KR816135	A4_GQ331047_Belgium

				A_KR816137	quasi-A3_ AM180624_Cameroon
				A_KR816138	B2_EU139543_China
				A_KR816139	C1_AB031265_Vietnam
				A_KR816142	D1_AJ344116_France
				A_KR816143	D2_AY090453_Sweden
				A_KR816145	D3_U95551_USA
				A_KR816146	D4_AB048703_Australia
				A_KR816147	D5_GQ205377_India
				A_KR816150	D-E_FN594770_Niger
				A_KR816152	E_FN545821_Nigeria
				A_MK127853	F2a_X69798_Brazil
				A_MK127854	G_EU833890_Canada
				A_MK127855	H_AY090454_Nicaragua
				A_MK127856	
				A_MK127857	
				D6_JQ927384	

[a]The GenBank accession number for study sequences is provided in parentheses following the study ID code. [b]HBV genotype or subgenotype is given followed by the GenBank accession number and geographic origin of the reference sequence.
